# Prognostic and immune regulating roles of YIF1B in Pan-Cancer: a potential target for both survival and therapy response evaluation

**DOI:** 10.1042/BSR20201384

**Published:** 2020-07-23

**Authors:** Jun Liu, Zheng Chen, Pingsen Zhao, Wenli Li

**Affiliations:** 1 Department of Clinical Laboratory, Yue Bei People’s Hospital, Shantou University Medical College, Shaoguan, Guangdong, P.R. China; 2 Liver Cancer Institute, Zhongshan Hospital, Fudan University and Key Laboratory of Carcinogenesis and Cancer Invasion, Ministry of Education, Shanghai, P.R. China; 3 Reproductive Medicine Center, Yue Bei People’s Hospital, Shantou University Medical College, Shaoguan, Guangdong, P.R. China

**Keywords:** HCC, ICGC, pan-cancer, TCGA, YIF1B

## Abstract

The neurotransmitter, serotonin has emerged as a tumor growth factor and immune response regulator through complex signaling pathways. Yip1 Interacting Factor Homolog B (YIF1B) is a membrane protein involved in serotonin receptor (HTR) membrane trafficking and signal transmission in neuropathy. Participation of YIF1B in serotonin-induced tumor growth and immune regulation has not been previously investigated. Data for analysis of YIF1B mRNA expression were downloaded from the website portals: The Cancer Genome Atlas (TCGA), GTEx, Cancer Cell Line Encyclopedia (CCLE) and International Cancer Genome Consortium (ICGC), including clinical and mutational information. Survival analysis included the Kaplan–Meier method for calculation of the cumulative incidence of the survival event and the log rank method for comparison of survival curves between groups. Infiltration levels of immune cells were calculated and correlated with YIF1B expression using the Spearman correlation test to evaluate significance. Further correlation analyses between YIF1B expression and mutation indicators such as tumor mutation burden (TMB), microsatellite instability (MSI), and mismatch repair (MMR) were also examined by the Spearman test. YIF1B expression was elevated in most cancer types and this high expression was indicative of poor overall survival (OS) and death-specific survival. In breast invasive carcinoma (BRCA) and liver hepatocellular carcinoma (LIHC), high YIF1B expression correlated with a poor disease-free interval (DFI), indicating a role in malignancy progression. There was a positive relationship between YIF1B expression and immune cell infiltration in several cancer types, and YIF1B also positively correlated with TMB, MSI, and methylation in some cancer types, linking its expression to possible evaluation of therapy response. The bioinformatics analyses have, therefore, established YIF1B as a good biomarker for prognostic and therapeutic evaluation.

## Introduction

Serotonin, a known neurotransmitter, has recently emerged as a tumor growth factor for several human cancers through interaction with its receptors (5-HTR 1–7), which are widely expressed across a range of tissues [1,2]. Furthermore, the expression of 5-HTRs in immune cells has identified a role for serotonin in regulation of both innate and adaptive immune functions, such as inflammation and wound healing. Some drug targeting experiments have implicated serotonin in pathological autoimmune diseases and cancer, whereby it affects cell differentiation, tumor cell migration and metastatic spread [3–7]. Serotonin-induced signaling is, however, extremely complex, as would be expected given the multiplicity of its receptors, which include members of the GPCR family and ligand-gated ion channels. Although specific roles of serotonin receptors have been investigated to some degree, including some drug targeting studies, it is likely that the tumorigenesis mechanisms will vary among different cancer types, their tissue origin and exposure factors [8–10].

Yip1 interacting factor homolog B (YIF1B) was first identified as a membrane traffic protein, involved in anterograde vesicle traffic between the endoplasmic reticulum and the Golgi apparatus, localizing mainly in the intracellular compartment [11]. YIF1B is found in a range of tissues and is proposed to shuttle between the organelle membranes of the protein secretory pathway. In neuronal dendritic cells, membrane-bound YIF1B directly associates with the C-terminal region of the 5-HT1A receptor, interfering with binding of serotonin to the distal part of the receptor. This observation has led to targeting of the process in development of treatments for depression [12,13]. Although HTRs are linked to tumorigenesis and immune microenvironment regulation, a role of YIF1B in these processes has not yet been explored. As a binding partner for HTRs, it is feasible that YTF1B could not only play a crucial role in serotonin-induced tumorigenesis, its differential binding could help rationalize the varied effects of serotonin on malignant changes observed among different cancer types. Here, we undertake a bioinformatics analysis to assess YIF1B expression in different tissues and its possible link to cancer. We found that YIF1B was highly expressed in almost all cancer types. The expression level was significantly correlated to survival, immune cell function and the mutation status of tumors. Also, YIF1B may influence methylation in some cancer types. The results identify YIF1B as a new prognostic marker for malignancy and an immune therapy response indicator in most cancer types. Furthermore, it may serve as a potential target for cancer therapy, especially in some low-response tumors.

## Materials and methods

### Data collection and processing

Pan-cancer sequencing data (Illumina platform) from The Cancer Genome Atlas (TCGA) database and Broad Institute Cancer Cell Line Encyclopedia (CCLE) database, as well as data linked to liver hepatocellular carcinoma (LIHC) from the International Cancer Genome Consortium (ICGC) database were extracted through their portal websites for analysis [14–16]. The whole data collection was filtered, removing missing and duplicated results, and transformed by log2(TPM +1), using the rma function within the R package (R studio version: 1.2.1335, R version: 3.6.1) [17,18]. Corresponding clinical information was also extracted through the portal websites, which included patients’ age, sex, tumor stages and clinical stages. Further, downloaded information, only available from TCGA database, was that of tumor mutation burden (TMB) and microsatellite instability (MSI). TMB was calculated as the total mutation incidences per million base pair, and MSI was counted by the number of insertion or deletion events that occurred in repeating sequences of genes.

### Cox regression analysis and survival analysis

Cox regression analysis was performed to examine the correlation between YIF1B expression and patients’ overall survival (OS), disease-specific survival (DSS) and disease-free interval (DFI) in each cancer type from the ICGC and TCGA databases in the R environment. The Kaplan–Meier method was used to construct the survival curves of patients in each cancer type after dividing patients into high and low YIF1B expression groups according to the best separation method. By deploying survivalROC and survival in the R package (rdocumentation.org/packages/survival), the specificity and time-dependent sensitivity of survival was analyzed [19]. The log-rank test was used to examine the difference between curves, and a *P*-value of less than 0.05 was considered significant.

### Immune cell infiltration enrichment

Tumor IMmune Estimation Resource (TIMER) is a database-derived web tool for immune cell infiltration calculation, which provides infiltration scores of six common types of immune cells, including B cells, CD4^+^ T cells, CD8^+^ T cells, macrophages, neutrophils and dendritic cells [20,21]. The immune cell infiltration scores of pan-cancer data from TCGA database have already been calculated using TIMER and are archived online. Here, the infiltration data were downloaded and used to test for correlation with YIF1B expression.

### Statistics

The Spearman Correlation test was used to assess the correlation between YIF1B expression and targets of interest, including immune cell infiltration scores (as described in the previous section for six immune cell types), TMB, MSI, mismatch repair (MMR) genes and methylation transferase genes. The comparison of YIF1B expression levels between groups, or between tumor and normal tissues, was performed with paired *t* tests or the *t* test, depending on whether the samples are paired or not. A *P*-value lower than 0.05 was considered significant. All graphs were generated through the R packages of ggplot2 and forestplot [22].

## Results

### YIF1B expression levels across various normal and cancer tissues

**Figure 1:**
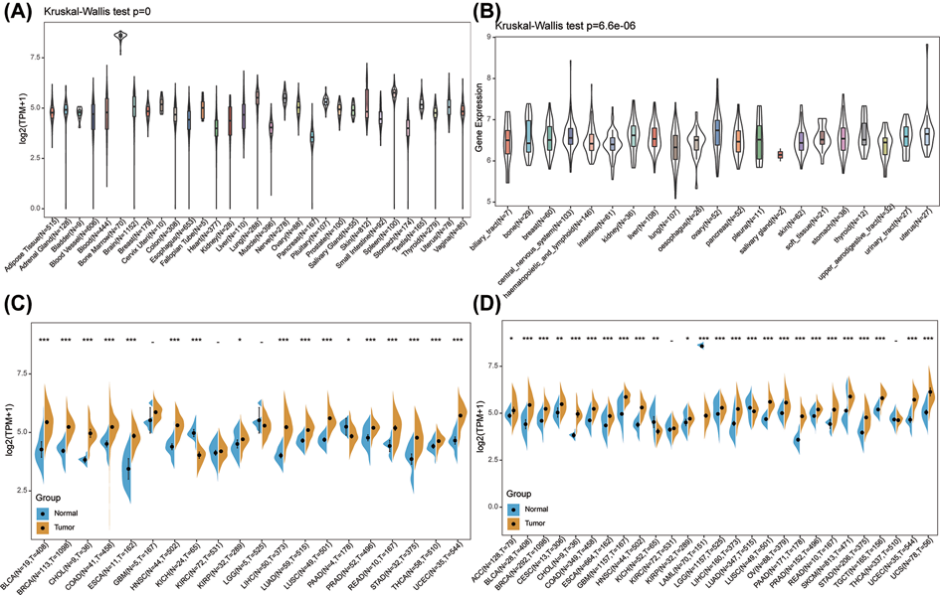
mRNA expression levels of YIF1B from different tissue origins and tumors (**A**) Normal mRNA expression levels of YIF1B in different tissues from GTEx database. (**B**) mRNA expression levels of YIF1B in various tumor cell lines from CCLE database. (**C**) mRNA expression difference of YIF1B between tumor and peri-tumor samples from TCGA database. (**D**) mRNA expression difference of YIF1B between normal, peri-tumor and tumor samples, combining data from TCGA and GTEx databases.

Using data from the GTEx database comprising different tissues from healthy people, the mRNA expression levels of YIF1B were established as similar across all tissues (Figure 1A), with the obvious exception of bone marrow. Bone marrow is an actively differentiating tissue and higher expression levels are not unexpected. Of more significance, in different cancer cell lines from the CCLE database, not only are YIF1B expression levels elevated ubiquitously, but narrower ranges are exhibited compared with the range of expression in the normal tissues (Figure 1B). Further comparison between relatively normal tissues and respective tumors showed YIF1B was highly expressed in most tumors, apart from the kidney chromophobe (KICH), which showed the reverse result with significance. Taking TCGA data alone, the expression difference achieved significance in 17 out of 20 cancer types (exceptions were the glioblastoma multiforme (GBM), kidney renal clear cell carcinoma (KIRC), and LGG cohorts). After combining the data from TCGA and GTEx, differences were significant for 25 out of 27 cancers. The KIRC and THCA cohorts showed similar expression levels between tumor and normal tissues, and KICH and acute myeloid leukemia (LAML) tumor tissues had decreased expression of YIF1B compared with normal tissues (Figure 1C,D).

### Analysis of link between YIF1B expression level and prognosis

**Figure 2:**
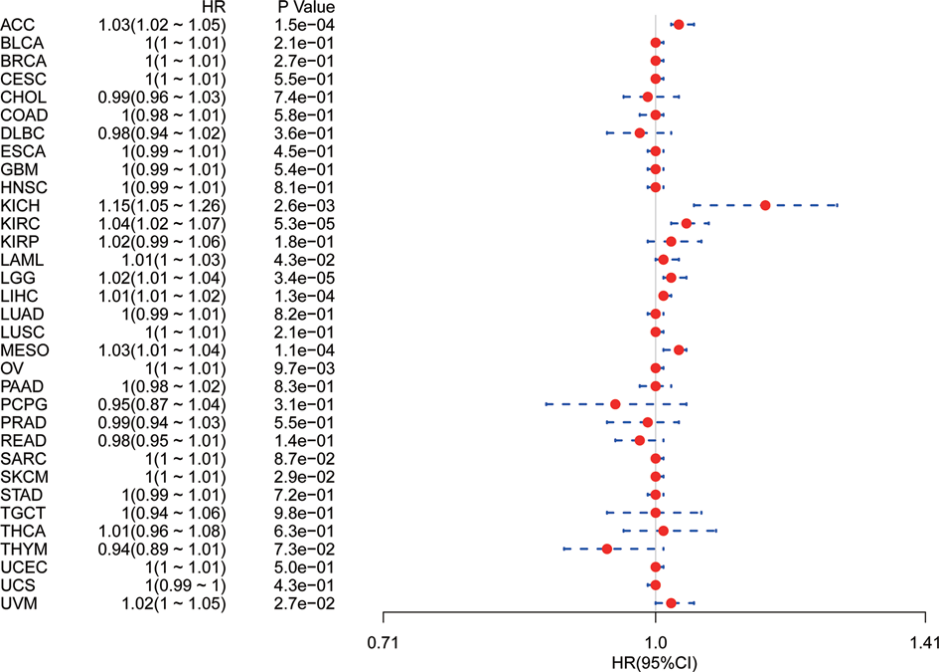
Association between mRNA expression levels of YIF1B and OS in multiple tumors from TCGA database Cox regression analysis, *P*<0.05 was considered significant. Abbreviations: BLCA, bladder urothelial carcinoma; CESC, cervical and endocervical cancers; CHOL, cholangiocarcinoma; COAD, colon adenocarcinoma; DLBC, lymphoid neoplasm diffuse large B-cell lymphoma; ESCA, esophageal carcinoma; HNSC, head and neck squamous cell carcinoma; KIRP, kidney renal papillary cell carcinoma; LUAD, lung adenocarcinoma; LUSC, lung squamous cell carcinoma; PAAD, pancreatic adenocarcinoma; PCPG, pheochromocytoma and paraganglioma; PRAD, prostate adenocarcinoma; READ, rectum adenocarcinoma; SARC, sarcoma; STAD, stomach adenocarcinoma; STES; stomach and esophageal carcinoma; TGCT, testicular germ cell tumor; THCA, thyroid carcinoma; THYM; thymoma; UCEC, uterine corpus endometrial carcinoma; UCS, uterine carcinosarcoma.

**Figure 3:**
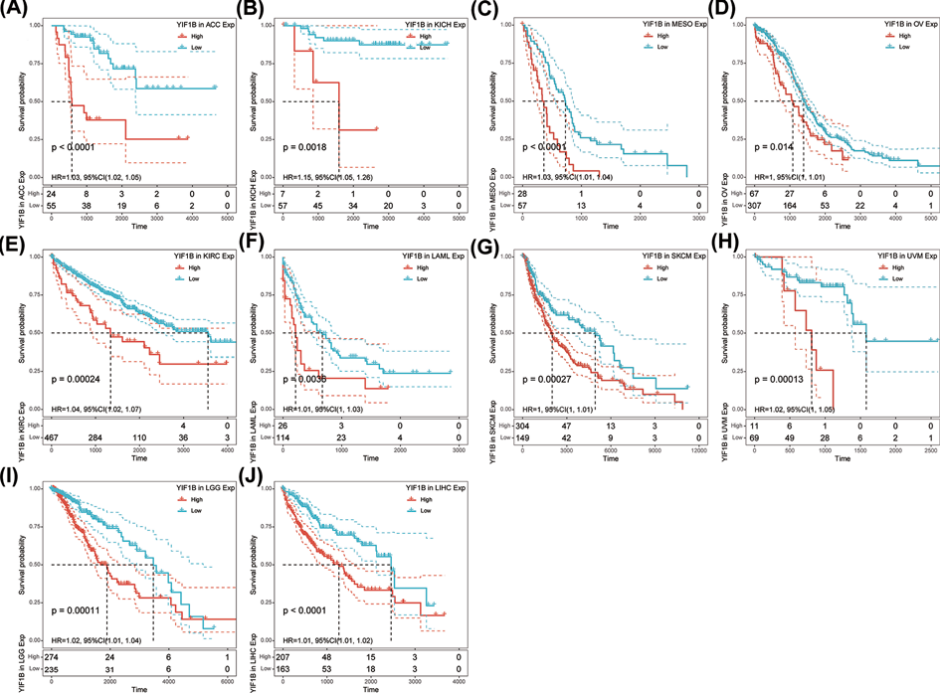
OS difference between high and low YIF1B mRNA expression groups (dichotomized by median expression) in significant prognosis-related tumors from TCGA database (**A**) OS difference between groups in ACC. (**B**) OS difference between groups in KICH. (**C**) OS difference between groups in MESO. (**D**) OS difference between groups in OV. (**E**) OS difference between groups in KIRC. (**F**) OS difference between groups in LAML. (**G**) OS difference between groups in SKCM. (**H**) OS difference between groups in UVM. (**I**) OS difference between groups in LGG. (**J**) OS difference between groups in LIHC. *P*<0.05 was considered significant, dash lines for 95% CI.

Using single variate Cox regression analysis, we assessed correlation between the respective expression level of YIF1B and OS in different cancer types, using data from TCGA database. The hazard ratios for YIF1B were significant for adrenalcortical carcinoma (ACC), KICH, KIRC, LAML, brain lower grade glioma (LGG), LIHC, mesothelioma (MESO), ovarian serous cystadenocarcinoma (OV), skin cutaneous melanoma (SKCM) and uveal melanoma (UVM), among which YIF1B had the highest risk effect in KICH (Figure 2). The following survival analyses, using patient data dichotomized for the median expression value in each cancer type (Figure 3), show that survival differences were all significant in OS-related cancer types, and that patients with high expression of YIF1B had worse outcomes (Figure 3).

**Figure 4:**
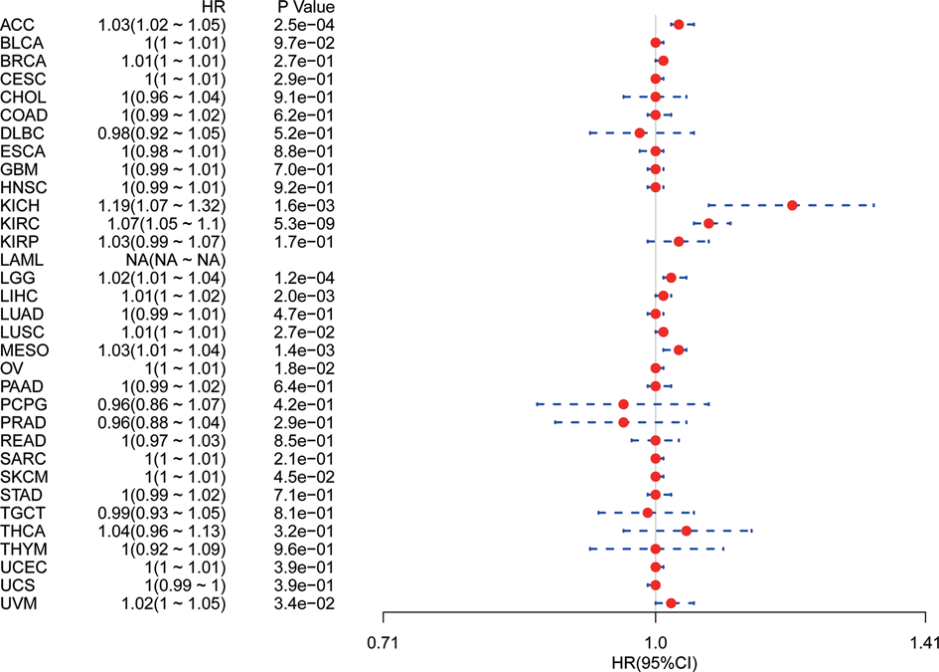
Association between YIF1B mRNA expression levels and DSS in multiple tumors from TCGA database Cox regression analysis, *P*<0.05 was considered significant. Abbreviations: BLCA, bladder urothelial carcinoma; BRCA, breast invasive carcinoma; CESC, cervical and endocervical cancers; CHOL, cholangiocarcinoma; COAD, colon adenocarcinoma; DLBC, lymphoid neoplasm diffuse large B-cell lymphoma; ESCA, esophageal carcinoma; HNSC, head and neck squamous cell carcinoma; KIRP, kidney renal papillary cell carcinoma; LUAD, lung adenocarcinoma; PAAD, pancreatic adenocarcinoma; PCPG, pheochromocytoma and paraganglioma; PRAD, prostate adenocarcinoma; READ, rectum adenocarcinoma; SARC, sarcoma; STAD, stomach adenocarcinoma; STES, stomach and esophageal carcinoma; TGCT, testicular germ cell tumor; THCA, thyroid carcinoma; THYM, thymoma; UCEC, uterine corpus endometrial carcinoma; UCS, uterine carcinosarcoma.

**Figure 5:**
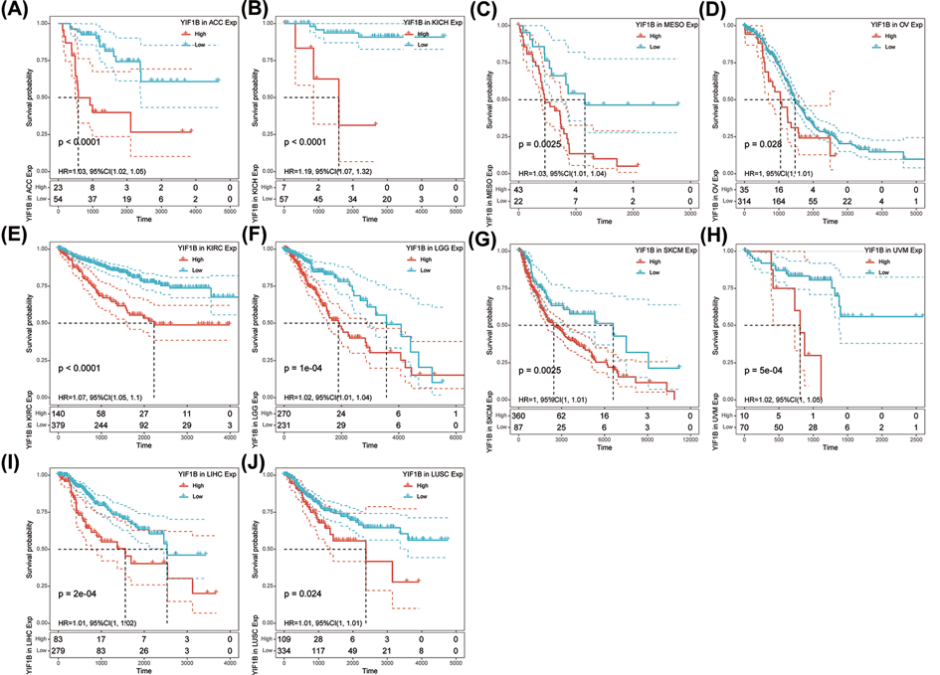
DSS difference between high and low YIF1B mRNA expression groups (dichotomized by median expression) in significant prognosis-related tumors from TCGA database (**A**) DSS difference between groups in adrenocortical carcinoma (ACC). (**B**) DSS difference between groups in KICH. (**C**) DSS difference between groups in MESO. (**D**) DSS difference between groups in ovary serous cystuadenocarcinoma (OV). (**E**) DSS difference between groups in KIRC. (**F**) DSS difference between groups in LGG. (**G**) DSS difference between groups in SKCM. (**H**) DSS difference between groups in UVM. (**I**) DSS difference between groups in LIHC. (**J**) DSS difference between groups in LUSC. *P*<0.05 was considered significant, dash lines for 95% CI.

It is possible, however, that OS could be affected by non-cancer related deaths during the follow-up period. We therefore re-analyzed the data for correlation between DSS and YIF1B expression among different cancers. The results of the Cox regression analysis gave similar results to those correlating to OS. Differences included identification of a significant risk effect for lung squamous cell carcinoma (LUSC) (in addition to the formerly mentioned ten types of cancers) (Figure 4) and an inability to calculate a hazard ratio for YIF1B in LAML due to lack of related data. In the following survival analysis, cancer types with high YIF1B expression again exhibited a worse prognosis in comparison with the low expression groups (Figure 5).

**Figure 6:**
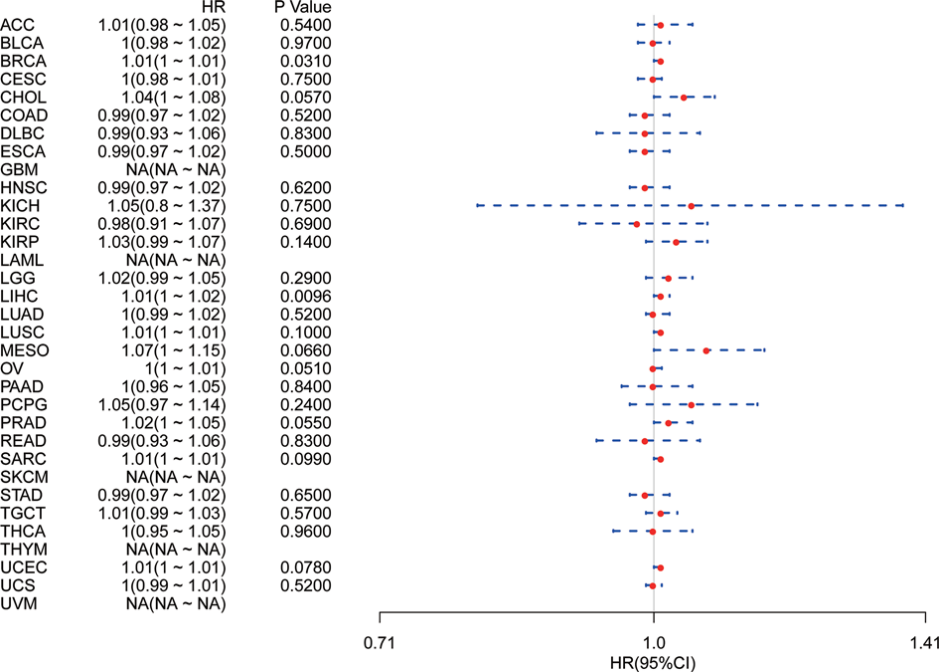
Association between mRNA expression levels of YIF1B and DFI in multiple tumors from TCGA database Cox regression analysis, *P*<0.05 was considered significant. Abbreviations: BLCA, bladder urothelial carcinoma; CESC, cervical and endocervical cancers; CHOL, cholangiocarcinoma; COAD, colon adenocarcinoma, DLBC, lymphoid neoplasm diffuse large B-cell lymphoma; ESCA, esophageal carcinoma; HNSC, head and neck squamous cell carcinoma, KIRP, kidney renal papillary cell carcinoma; LUAD, lung adenocarcinoma; PAAD, pancreatic adenocarcinoma; PCPG, pheochromocytoma and paraganglioma; PRAD, prostate adenocarcinoma; READ, rectum adenocarcinoma; SARC, sarcoma; STAD, stomach adenocarcinoma; STES, stomach and esophageal carcinoma, TGCT, testicular germ cell tumor; THCA, thyroid carcinoma; THYM, thymoma; UCEC, uterine corpus endometrial carcinoma; UCS, uterine carcinosarcoma.

**Figure 7:**
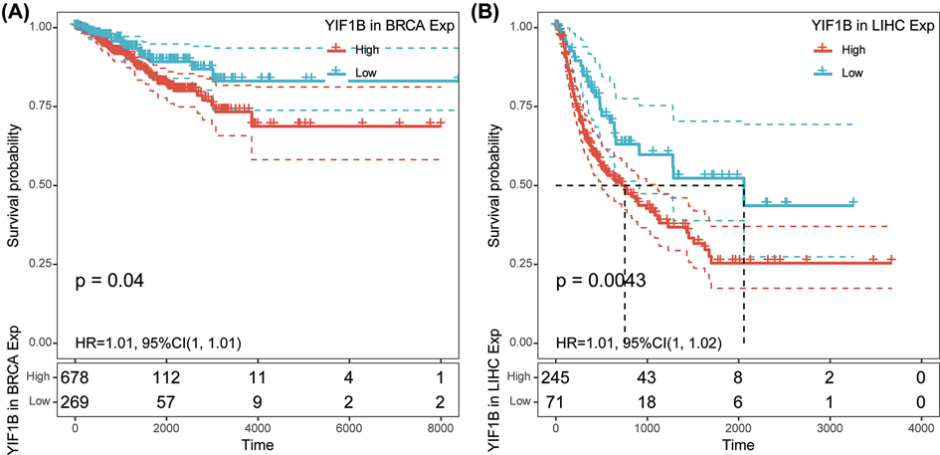
DFI difference between high and low YIF1B mRNA expression groups (dichotomized by median expression) in BRCA and LIHC from TCGA database (**A**) DFI difference between groups in BRCA. (**B**) DFI difference between groups in LIHC. *P*<0.05 was considered significant, dash lines for 95% CI.

Finally, correlation between YIF1B expression and DFI was also analyzed by Cox regression. Significant hazard ratios were obtained for breast invasive carcinoma (BRCA) and LIHC (Figure 6). After separating patients into two groups according to the respective median YIF1B expression for the cancer type, survival differences were significant between high and low expression groups. In both BRCA and LIHC, high expression patients exhibited early recurrence after tumor resection. For LIHC in particular, the significance of YIF1B in cancer progression is highlighted by the gap of over 1000 days between survival times (with respect to DFI) of high and low expression groups (Figure 7).

### YIF1B may regulate the tumor immune microenvironment by influencing immune infiltration in various cancer types

**Figure 8:**
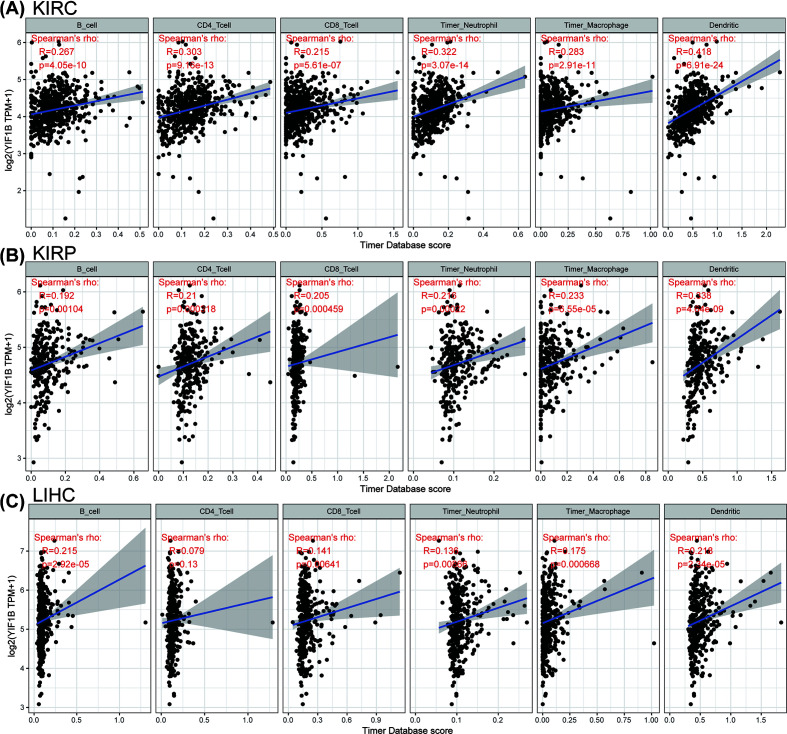
Top-ranked correlation between YIF1B mRNA expression level and infiltration scores of six common-type immune cells (B cell, CD4^+^ T cell, CD8^+^ T cell, neutrophil, macrophage, dendritic cell), calculated by TIMER from TCGA database (**A**) Correlation between six immune cell infiltration scores and YIF1B mRNA expression in KIRC. (**B**) Correlation between six immune cell infiltration scores and YIF1B mRNA expression in KIRP. (**C**) Correlation between six immune cell infiltration scores and YIF1B mRNA expression in LIHC. Spearman correlation test, *P*<0.05 was considered significant.

YIF1B is known to participate in maintenance of Golgi architecture in tumor cells, and may thus affect immune cell differentiation through the serotonin pathway. To assess whether the immune microenvironment of tumors could be influenced by this pathway, we tested the correlation between YIF1B expression and the level of immune cell infiltration in each cancer type. Using the infiltration scores of six immune cell types (B cell, CD4^+^ T cell, CD8^+^ T cell, neutrophil, macrophage and dendritic cell) available in the TIMER database, derived from TCGA, we found that there was indeed a significant correlation in several tumors. The three top-ranking tumor cohorts were KIRC, kidney renal papillary cell carcinoma (KIRP) and LIHC. Their corresponding linear regression graphs show that high YIF1B expression is linked to a possible increased infiltration level by immune cells. It is worth noting that, among all cell types in these three cancers, dendritic cells had the highest significant coefficients (Figure 8).

### Correlation of YIF1B expression with expression of some immune checkpoint genes for certain cancers implicates YIF1B in the tumor immune response

**Figure 9:**
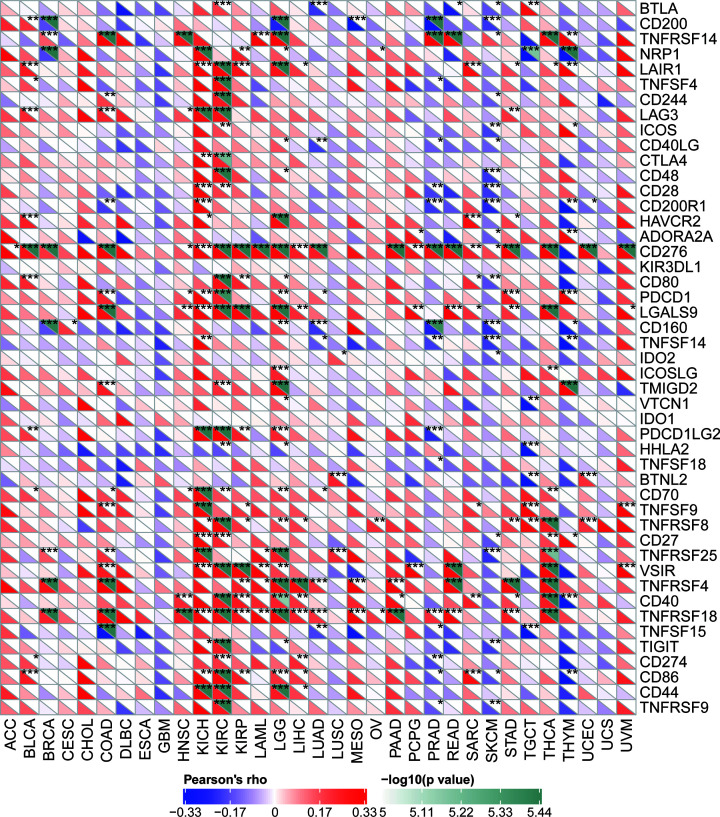
Correlation between YIF1B mRNA expression levels and acknowledged immune checkpoints’ mRNA expression in multiple tumors from TCGA database The lower triangle in each tile indicates coefficients calculated by Pearson’s correlation test, and the upper triangle indicates log10 transformed *P*-value. *P<0.05, **P<0.01, ***P<0.001.

There are now several genes strongly associated with and recognized as checkpoint components in the immune response. The mRNA sequence databases enabled us to evaluate whether a link between YIF1B expression and expression of such checkpoint genes exists. Correlation analyses between YIF1B and checkpoint gene expression found a high correlation (*P*<0.05) with tumor necrosis factor (TNF)-related immune genes (TNFRSF4, 8, 14, 18) and CD276 (B7H3) in various cancer types. Furthermore, in KICH and KIRC, significant co-expression of YIF1B with more immune checkpoint genes was detected. The results, particularly for KICH and KIRC, implicate YIF1B in regulation of the tumor immune response through modulation of immune checkpoint activity. It is also interesting that in esophageal carcinoma (ESCA), GBM, prostate adenocarcinoma (PRAD), SKCM and thymoma (THYM), expression of YIF1B was negatively correlated with most immune checkpoint molecules though, for some of them, not to a significant degree (Figure 9).

### YIF1B is associated with the TMB and MSI in some cancers

**Figure 10:**
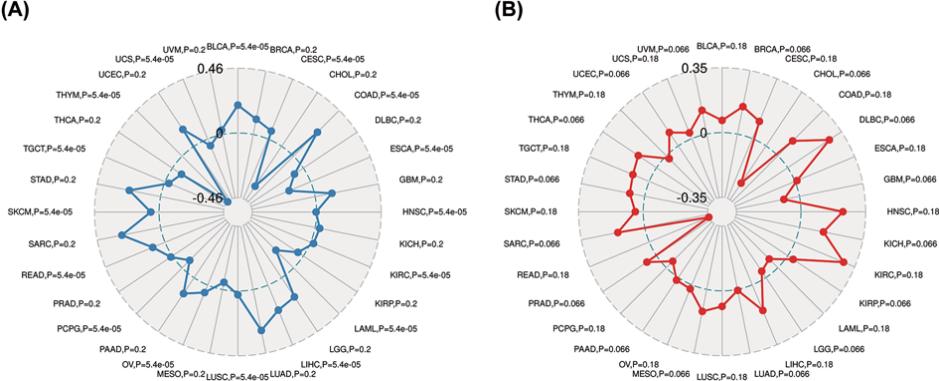
Relation between TMB, MSI and YIF1B mRNA expression levels in various tumors in TCGA database TMB was counted by total mutation incidences per million basepair in each tumor, and MSI was counted by total incidence of deletion or insertion in repeating sequences per million basepair. (**A**) Correlation between TMB and YIF1B expression. (**B**) Correlation between MSI and YIF1B expression. Spearman correlation test, *P*<0.05 was considered significant.

TMB and MSI are valid prognostic biomarkers and immune therapy response indicators in many kinds of tumor. We examined their respective relationships with YIF1B expression in various cancer types in order to investigate a link between YIF1B activity and mutation in specific cancer types. The correlation between YIF1B expression and TMB achieved significance (*P*<0.05) in 16 out of 32 cancer types for which data were available (colon adenocarcinoma (COAD), bladder urothelial carcinoma (BLCA), LIHC, rectum adenocarcinoma (READ), cervical and endocervical cancers (CESC), OV, SKCM, KIRC, LUSC, head and neck squamous cell carcinoma (HNSC), testicular germ cell tumor (TGCT), uterine carcinosarcoma (UCS), pheochromocytoma and paraganglioma (PCPG), ESCA, LAML and THYM), of which COAD, BLCA and LIHC had the highest coefficients, while THYM, LAML and ESCA had the lowest coefficients (Figure 10A). The coefficient values would indicate that YIF1B expression positively correlates with high mutation status in COAD, BLCA and LIHC, but low mutation in THYM, LAML and ESCA (particularly THYM). Correlation of YIF1B expression with MSI was tested in 32 cancer types, of which none achieved significance. Given that the data volumes for MSI were low, it is worthwhile considering results that are close to the significance threshold, which applied to 16 of the cancer types (lymphoid neoplasm diffuse large B-cell lymphoma (DLBC), KICH, sarcoma (SARC), BRCA, MESO, THCA, UVM, uterine corpus endometrial carcinoma (UCEC), stomach adenocarcinoma (STAD), PRAD, KIRP, pancreatic adenocarcinoma (PAAD), lung adenocarcinoma (LUAD), LGG, GBM and cholangiocarcinoma (CHOL): *P*=0.066) (Figure 10B). Among those cancer types, DLBC, KICH and SARC had the highest coefficients, demonstrating a positive correlation between YIF1B expression and MSI. In contrast, the highest absolute value of coefficient obtained for CHOL, demonstrated a significant negative correlation between YIF1B expression and MSI. It is notable that the absolute coefficients for correlation with TMB or MSI were both relatively high for the LIHC and CHOL cohorts compared with other cancer types, although overall the number of cancer types showing significant correlation to these indicators of mutation was relatively low.

### YIF1B expression is strongly related to MMR defects in different cancers and may interfere with methylation after transcription

**Figure 11:**
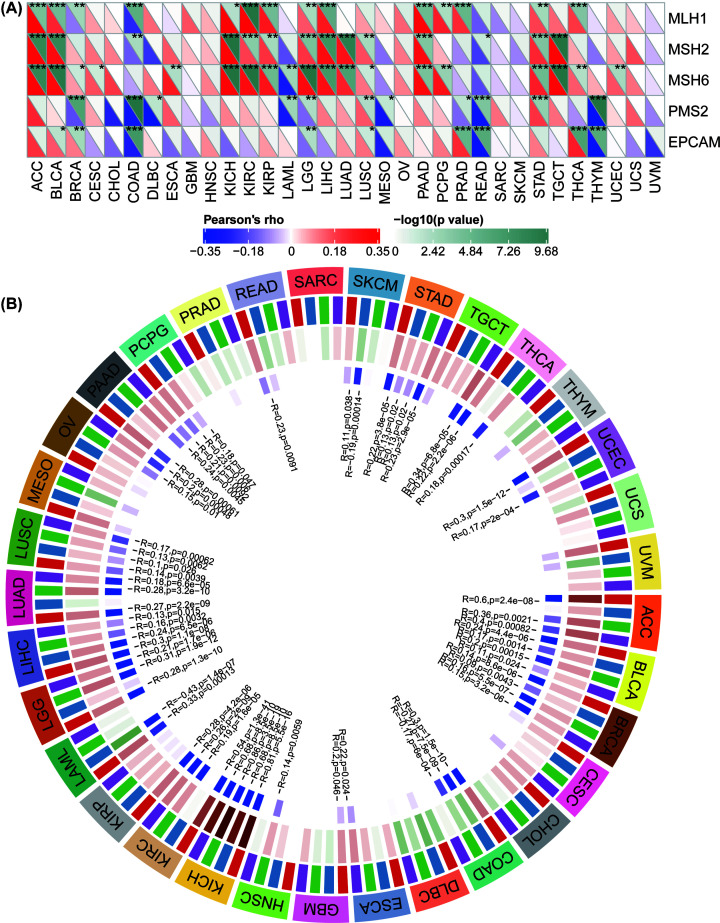
Relationship between MMR defects, methyation levels and YIF1B mRNA expression level in various tumors in TCGA database (**A**) Correlation between YIF1B mRNA expression and mutation levels of five significant MMR genes (*MLH1, MSH2, MSH6, PMS2, EPCAM*). The lower triangle in each tile indicates coefficients calculated by Pearson’s correlation test, and the upper triangle indicates log10 transformed *P*-value. *: *P*<0.05, **: *P*<0.01, ***: *P*<0.001. (**B**) Correlation between YIF1B and four methyltransferases (DNMT1: red, DNMT2: blue, DNMT3A: green, DNMT3B: purple) mRNA levels.

Having established a correlation between YIF1B expression and the mutation indicators, TMB and MSI, further investigation of links between YIF1B expression and tumorigenesis mechanisms was warranted, in particular a relationship with MMR defects and methylation of specific tumor suppression genes. We thus examined the relationship between YIF1B expression and some well-established MMR genes (*MLH1, MSH2, MSH6, PMS2* and *EPCAM*). Accordingly, the results show that in broad cancer types (ACC, BLCA, BRCA, COAD, KICH, KIRC, KIRP, LGG, LIHC, LUSC, PAAD, PRAD, PEAD, STAD and THCA), YIF1B expression significantly and highly correlates with expression of MMR genes; *MLH1, MSH2* and *MSH6* all positively correlated with YIF1B in most of these cancer types. Interestingly, in cancer types of colon origin (READ and COAD), YIF1B expression clearly negatively correlates with all five MMR genes, indicative of a potential role for MMR regulation in colon tumorigenesis (Figure 11A).

With respect to correlation of YIF1B expression with that of four methylation transferases (DNMT1, DNMT2, DNMT3A, DNMT3B), the results show that there is co-expression in specific cancer types (KICH, KIRC, ACC, BRCA, BLCA, COAD, LUSC, LUAD, LIHC, LGG, LAML, PAAD, OV, PCPG, SKCM, STAD, TGCT, THCA, UCEC). Of note, tumors of the kidney and adrenal gland (KICH, KIRC and ACC) demonstrated significantly high co-expression coefficients, which were above 0.5, while coefficients for other cancer types were all lower (Figure 11B).

### YIF1B expression is significant in LIHC

Considering all results thus far, we identified a correlation between YIF1B expression and many tumorigenesis and immune microenvironment regulating mechanisms, with LIHC giving significant coefficients in most analyses. To further explore the potential of YIF1B as a novel biomarker for LIHC, a cancer for which early detection and prognosis are particularly poor, we undertook further analyses using data from the ICGC. In comparison with paired or unpaired normal liver tissues, LIHC exhibits higher expression of YIF1B (Supplementary Figure S1A,B). After dividing patients with LIHC into risk groups according to their clinical stages, expression of YIF1B increased with escalation of clinical stage, demonstrating that expression of YIF1B correlated to disease progression in LIHC patients (Supplementary Figure S1C). In follow-on survival analysis, after dichotomizing patients according to their mean YIF1B expression value, patients in the high expression group had worse OS, which is consistent with in the results obtained using TCGA data (Supplementary Figure S1).

## Discussion

Our results have shown that YIF1B is expressed widely among different tissues, and expression is particularly high in bone marrow. It is possible that high cell proliferation and turnover may require high YIF1B levels, which would explain higher expression in bone marrow. When comparing tumors with corresponding normal tissues, YIF1B expression was elevated in different cancer types, and this high expression was related to worse OS and death-specific survival in those cancer types. An exception to the rule was KICH, which demonstrated significant decreased levels of YIF1B. A correlation with disease progression rates was identified for LIHC and BRCA, for which patients with high YIF1B expression suffered from early recurrence of tumor. The progression rate was particularly enhanced in LIHC. These are important results with respect to a central role for YIF1B in serotonin-induced signaling pathways. Previous research has shown that YIF1B is involved in anterograde vesicle traffic in cells, transporting ‘cargo’ proteins (including the serotonin receptor HTR1A) from the endoplasmic reticulum to the cell membrane via the Golgi apparatus; such cell membrane localization being accelerated upon knocking out YIF1B in HeLa cells [11]. YIF1B also influences the integrity of Golgi apparatus in the long term, which supports the proposal that it localizes mainly outside the Golgi and shuttles between membrane locations. Although YIF1B is known to interact with the serotonin receptor, HTR1A, the identification of further binding partners and the full extent of its involvement in serotonin signaling requires further investigation. A link to signaling pathways via HTR receptors is the likely reason for association of YIF1B mutations with functional changes to specific proteins in neuronal cells, causing encephalopathy, epilepsy and movement disorder [23]. Although serotonin has been linked to cancer, a role for YIF1B in tumorigenesis and cancer progression has not been previously established. However, the correlation discovered here between YIG1B expression and cancer in various tissues, including a clear link to cancer survival and prognosis, make it an interesting biomarker for cancer monitoring and further investigation.

In our correlation analysis, expression of YIF1B was clearly also related to immune cell infiltration of different tumors, the highest scoring cancer types being KIRC, KIRP and LIHC. Of the six immune cell types, dendritic cells gave the highest coefficients. Our results are consistent with previous studies, in which serotonin receptors (HTRs) have been shown to be widely expressed in peripheral immune cells [3,5,24]. In particular, YIF1B has been shown to facilitate downstream signaling after combination of serotonin and HTR1A receptor in dendritic cells [12,13]. We propose that the immune cell infiltration data we observe, linked to expression of YIF1B, are related to serotonin’s function in immune cell recruiting and differentiation [7]. Of note, expression of YIF1B was also related to expression of some specific immune checkpoint genes across several tumors. Such checkpoint genes included CD276 (B7H3), CD40, TNFRSF14, TNFRSF4, TNFRSF18 and TNFRSF8. Up-regulation of such checkpoint genes may be the consequence of immune cell differentiation and polarization due to serotonin, or changes to protein trafficking caused by YIF1B up-regulation. However, considering the likely multiplicity of interactions YIF1B can make and its consequent diverse functional links, it is possible YIF1B can drive malignant changes through other undiscovered mechanisms [13,25,26].

We have also demonstrated a link between YIF1B expression and TMB and MSI in some cancer types, and certain tumors also exhibit co-expression of YIF1B with the major MMR genes and methylation transferases. Previous studies have correlated TMB and MSI to patients’ drug responses, especially for drugs that target immune checkpoint inhibitors, such as a TGF-β antagonist and PD-1 inhibitor [27–32]. We propose, therefore, that YIF1B could be deployed as an additional indicator for immune therapy evaluation of cancer patients after administration. It is interesting that MSI is now recognized as an indicator for differentiating the tumor type in COAD patients. Furthermore, COAD patients with high MSI have demonstrated better checkpoint inhibitor responses and survival in both low and high clinical stages [33–35]. Both TMB and MSI of COAD were positively related to YIF1B expression in our study, which would support our proposition that YIF1B might make a good indicator for potential drug responses (and MSI), certainly in COAD.

An overview of our results consistently links YIF1B expression to LIHC and indicates that YIF1B is of potential use in the prediction of LIHC prognosis and treatment. The link between expression of YIF1B and malignancy progression and recurrence in LIHC patients was consistent in different databases. Although much more information on the molecular role of YF1B in immune regulation is required to understand the strong correlation between YIG1B expression and LIHC, the liver is a highly metabolic organ with a different immune microenvironment, and more recent studies have described it as an immune suppressive organ [36,37]. In our results, YIF1B expression is highly related to immune cell infiltration in LIHC, as well as TMB, the MMR gene and methylation transferase expression. YIF1B may influence tumorigenesis in LIHC through many mechanisms, considering its anterograde membrane trafficking function and links to an immune response. Further studies should be conducted to determine the explicit mechanisms. Irrespective of the mechanism linking YIF1B expression to LIHC prognosis, the results offer up YIF1B as a possible additional diagnostic marker. YIF1B is also a possible additional therapeutic target for LIHC, a cancer with poor prognosis, particularly if identified late. One benefit of targeting YIF1B is that decreasing its activity may not interfere with the function of normal non-tumor cells. This proposal could perhaps be extrapolated to treatment of other low-response tumors [11].

Although our study has provided useful indication of involvement of YIF1B in tumorigenesis and regulation of the immune environment in tumor cells, it does comprise some limitations. First, as a purely bioinformatics analysis, it is completely dependent on information available in open-access databases, with no confirmation through experimentation. In our study, the evaluation of YIF1B expression is based entirely on mRNA levels reported in the mentioned databases, even though this might not reflect levels of functional protein. For example, protein activity might be affected in normal or cancer cells by post-transcription modification and/or regulated proteolysis. Future investigation will focus on validating our data by experiment and exploring possible mechanisms for YIF1B in tumorigenesis. Second, although previous studies have shown serotonin can influence the immune microenvironment of tumors, and YIF1B could influence the immune status of cancer patients by interfering with serotonin signaling, direct experimental demonstration of an effect of YIF1B on the immune microenvironment of a tumor is needed. Third, the link we have demonstrated between YIF1B expression and TMB, MSI and MMR genes lacks any mechanistic interpretation from supporting experimental data. Although it is possible that the processes of methylation and MMR are directly influenced by YIF1B activity in specific cancer types, much more experimental evidence would be required to prove this is the case.

## Conclusion

YIF1B is highly expressed in various tumors and this high expression is related to poor survival and disease progression, especially for LIHC. YIF1B expression also correlates with tumor infiltration by immune cells, immune checkpoint gene expression and immune therapy indicators, such as TMB, MSI, MMR genes and methylation genes. Taken together, the data indicate that YIF1B offers several cancers a valuable new biomarker for prognostic and immune therapy response evaluation.

## Supplementary Material

Supplementary Figure S1

## Data Availability

The datasets generated and/or analyzed during the current study are available in the TCGA (https://portal.gdc.cancer.gov/) and ICGC (https://icgc.org/) repository.

## Abbreviations

ACC: adrenalcortical carcinoma
BLCA: bladder urothelial carcinoma
BRCA: breast invasive carcinoma
CCLE: Cancer Cell Line Encyclopedia
CHOL: cholangiocarcinoma
COAD: colon adenocarcinoma
DFI: disease-free interval
DLBC: lymphoid neoplasm diffuse large B-cell lymphoma
DSS: disease-specific survival
DNMT: DNA methyltransferase
ESCA: esophageal carcinoma
GBM: glioblastoma multiforme
GTEx: The Genotype-Tissue Expression
HTR: serotonin receptor
ICGC: International Cancer Genome Consortium
KICH: kidney chromophobe
KIRC: kidney renal clear cell carcinoma
KIRP: kidney renal papillary cell carcinoma
LAML: acute myeloid leukemia
LGG: brain lower grade glioma
LIHC: liver hepatocellular carcinoma
LUAD: lung adenocarcinoma
LUSC: lung squamous cell carcinoma
MESO: mesothelioma
MMR: mismatch repair
MSI: microsatellite instability
OS: overall survival
OV: ovarian serous cystadenocarcinoma
PAAD: pancreatic adenocarcinoma
PCPG: pheochromocytoma and paraganglioma
PD-1: programmed cell death protein 1s
PRAD: prostate adenocarcinoma
READ: rectum adenocarcinoma
ROC: Receive Operating Characteristic
SARC: sarcoma
SKCM: skin cutaneous melanoma
STAD: stomach adenocarcinoma
TCGA: The Cancer Genome Atlas
TGCT: testicular germ cell tumor
THYM: thymoma
TIMER: Tumor IMmune Estimation Resource
TMB: tumor mutation burden
TNF: tumor necrosis factor
UCEC: uterine corpus endometrial carcinoma
UVM: uveal melanoma
YIF1B: Yip1 Interacting Factor Homolog B
