# Supplementary material for: Prognostic and immune regulating roles of YIF1B in Pan-Cancer: a potential target for both survival and therapy response evaluation
Source: Biosci Rep. 2020 Jul 23;40(7):BSR20201384. doi: 10.1042/BSR20201384 (PMC7378310; doi:10.1042/BSR20201384)
Supplement: Supplementary Figure S1 [file BSR-2020-1384_supp.pdf]

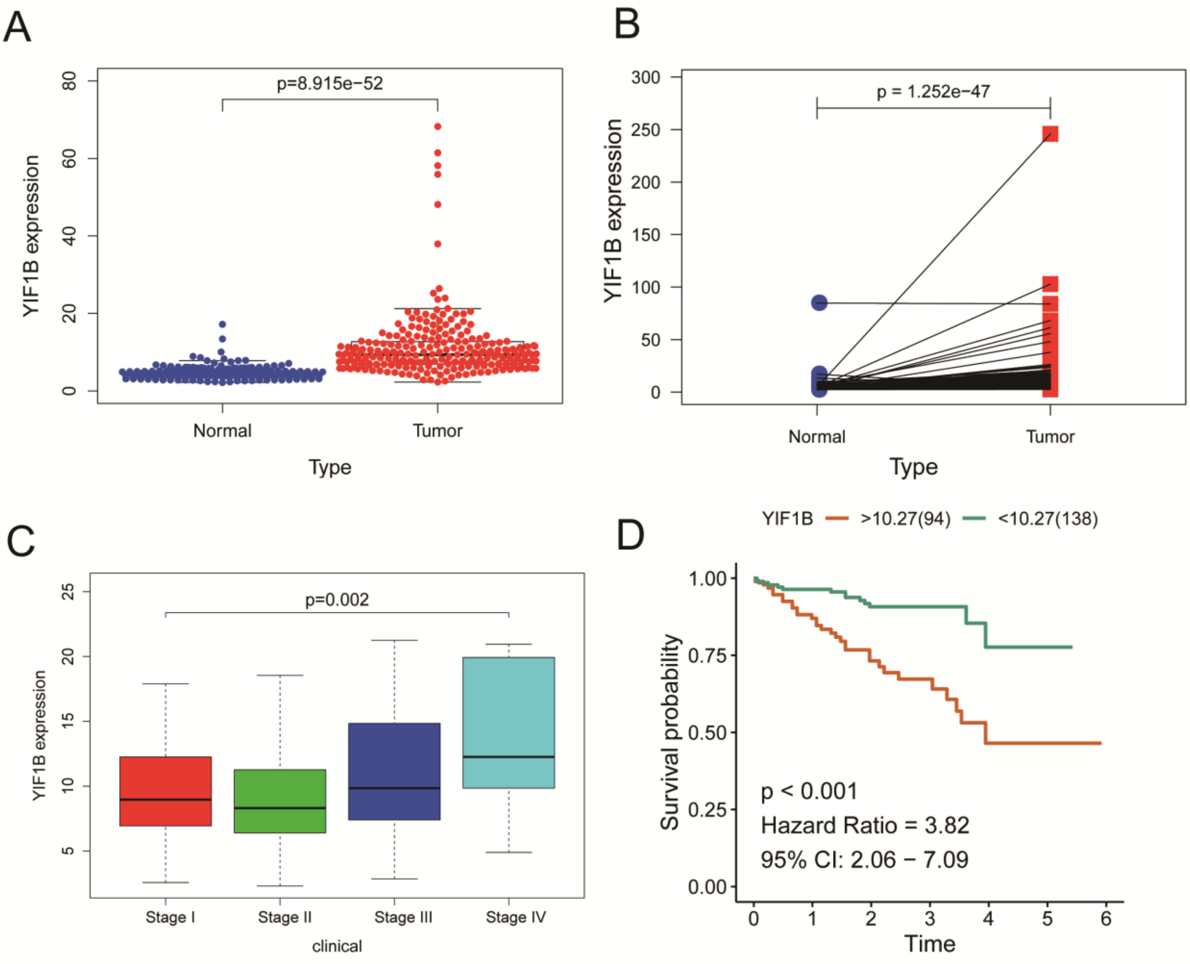

Supplementary Figure S1. YIF1B mRNA expression in liver hepatocellular carcinoma (LIHC) and relative normal liver tissues in ICGC database. a. Expression difference of YIF1B in LIHC and relatively normal tissues. b. YIF1B mRNA expression difference in paired LIHC and normal tissues. c. Expression levels of YIF1B in LIHC of different stages. d. Survival difference between high and low YIF1B expression groups, separated by median expression level. ($p< 0.05$ was significant).
